# Medial Patellofemoral Ligament Reconstruction: A Case Report for an Integrated Rehabilitation Approach

**DOI:** 10.7759/cureus.53137

**Published:** 2024-01-29

**Authors:** Abhishek Gujar, Pratik R Jaiswal, Swapnil U Ramteke

**Affiliations:** 1 Sports Physiotherapy, Ravi Nair Physiotherapy College, Datta Meghe Institute of Higher Education and Research, Wardha, IND

**Keywords:** excercise and weight training, arthroscopic repair, sports physiotherapy, physical therapy rehabilitation, medial patellofemoral ligament

## Abstract

Medial patellofemoral ligament (MPFL) reconstruction is a surgical treatment, primarily indicated for patients grappling with recurrent patellar instability stemming from traumatic injury or underlying anatomical anomalies. This abstract aims to elucidate the indispensable role of physiotherapy in the post-operative rehabilitation trajectory for individuals subjected to MPFL reconstruction. Physiotherapy emerges as a linchpin in securing a triumphant outcome, fostering the healing of patellar stability, augmentation of range of motion (ROM), and bolstering muscular strength while concurrently mitigating potential complications. The abstract accentuates salient facets of a physiotherapeutic regimen, encompassing prompt post-operative mobilization, meticulously tailored exercise paradigms, adept utilization of manual therapy modalities, and comprehensive patient education. Notably, this collaborative endeavor between orthopedic surgeons and physiotherapists is pivotal in optimizing patient convalescence, restoring them to their pre-injury functional acumen. A paramount emphasis is placed on individualized rehabilitation strategies, gradual and systematic exercise protocols, and patient adherence, thereby underscoring how the harmonious synergy between surgical and physiotherapeutic interventions augments the prospects of achieving a successful MPFL reconstruction outcome.

## Introduction

The medial patellofemoral ligament (MPFL) is a band-shaped anatomical structure that emanates proximally from the medial femoral epicondyle and extends toward the medial aspect of the patella, exhibiting variability in its dimensions, structural integrity, and overall length [1]. The patella, a triangular-shaped sesamoid bone, features its apex at the inferior pole, from where it gives rise to the patellar tendon, which attaches to the tibial tubercle. The superior pole of the patella constitutes the base and serves as the anchoring site for the quadriceps tendon. Functionally, the patella enhances the mechanical advantage of the quadriceps tendon by altering the angle at which it acts upon the femur [2]. The patella's dorsal aspect interfaces with the distal femur's anterior aspect, nestled within the trochlear groove. This surface is divided into distinct medial and lateral facets, each engaging with their counterparts on the medial and lateral femoral condyles [3]. The peri-patellar soft tissues, notably the MPFL and the vastus medialis oblique (VMO) muscle, play a substantial role in ensuring joint stability [4].

Acute patellar dislocation is a frequently occurring injury across various activities. Studies on non-surgical interventions indicate that 17% to 44% of patients encounter recurrent dislocations. The MPFL constitutes the superior boundary of the second anatomical layer and is situated deep in the distal muscle belly of the VMO [5]. Lateral dislocation of the patella is a prevalent knee injury among the youthful, physically active demographic. Indeed, earlier investigations have determined that acute patellar dislocation ranges from 23 to 77 cases per 100,000 person-years, contingent on the demographic profile under scrutiny. The zenith of incidence, projected at 148 cases per 100,000 person-years, is observed within the age bracket of 14 to 18 years [6,7]. The MPFL represents the primary anatomical structure frequently affected in cases of lateral patellar dislocation. Rupture of the MPFL constitutes approximately 3% of total knee injuries. The surgical procedure known as MPFL reconstruction is a dependable method with favorable outcomes; however, it exhibits varying rates of recurrent instability [8].

MPFL reconstruction exhibits a reduction in recurrent dislocation when compared to MPFL repair or nonoperative interventions. However, it does come with an elevated likelihood of complications. On the other hand, MPFL repair demonstrates a decrease in postoperative redislocation compared to nonoperative treatment, though it does not manifest a clear advantage in the context of primary dislocation [9]. Extensive follow-up data reveal that patients subjected to nonoperative management for patellar dislocation generally exhibit satisfactory outcomes compared to individuals in analogous prior investigations. However, significant disparities in final results emerge based on the treatment approach. An analysis of the long-term progression of 100 patients underscores that over 50% experienced symptoms, such as recurrence or subsequent issues, after a 13-year follow-up period. This proportion is deemed excessively high and needs to meet acceptable standards [10]. The mean patient-reported satisfaction rate reached 96%. In suitable acute patellar dislocation, we advocate the primary repair of the MPFL and the VMO muscle. This approach is advised to mitigate the risk of recurrent dislocation, chronic subluxation, as well as the associated pain and disability [11].

From a biomechanical perspective, the patellofemoral joint represents one of the intricately designed human articulations. It frequently serves as a prominent etiological factor for discomfort among physically active adult and adolescent populations, with a notable preponderance in the female demographic. Patellofemoral disorders constitute a substantial proportion, ranging between 20% and 40%, of the overall caseload of knee-related ailments observed in family medicine, sports medicine, and orthopedic clinical settings [12-14].

## Case presentation

Patient information

While bating the ball, a 19-year-old male patient cricket player hits his knee. However, he continued to play. After three days, while batting, he was returning from the front foot to the back foot position when the dislocation of his patella occurred; he started having pain in his right knee, which was insidious in onset and nonprogressive in nature, non-radiating and increased while walking. After two months, the patient started having instability while walking, due to which the patient had two to three episodes of falls. The patient complained of a few episodes of locking of the right knee while walking. On radiological investigation, he was diagnosed with MPFL tear and suggested for arthroscopic repair. After the arthroscopic repair, the patient was having difficulty in walking for which he was referred to sport physiotherapy rehabilitation.

Clinical findings

The right knee patient was examined in a supine position with both anterior superior iliac spine (ASIS) at the same level and in sitting and standing position examination of both knees. On inspection, the scar was seen over the medial aspect of the knee; swelling was present, and joint mobility was incomplete and painful. Pain on the visual analog scale (VAS) on activity was 7, on rest was 2, and the nature of pain was on and off.

Radiological investigation

**Figure 1:**
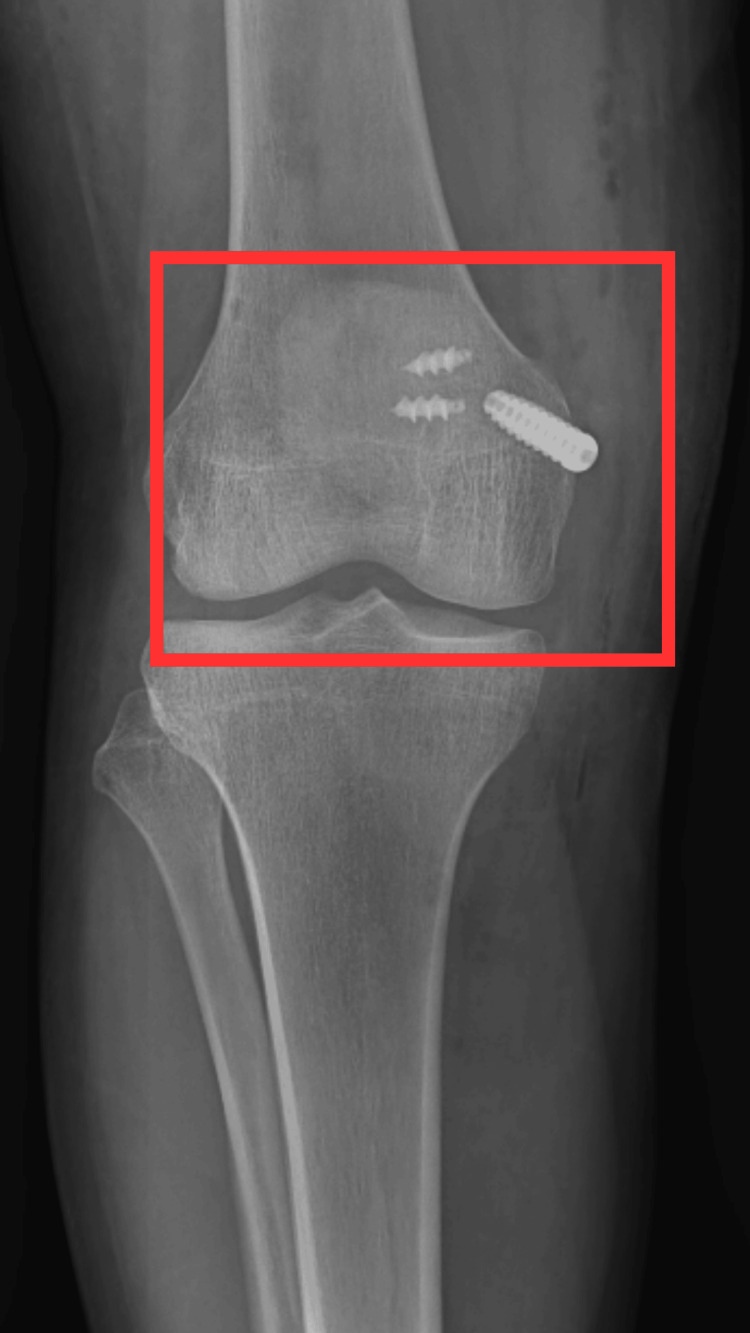
Post-operative x-ray of right knee joint

A radiological investigation like an x-ray was carried out after the operation. Figure 1 shows the post-operative x-ray of the patient with MPFL reconstruction.

Management

**Figure 2:**
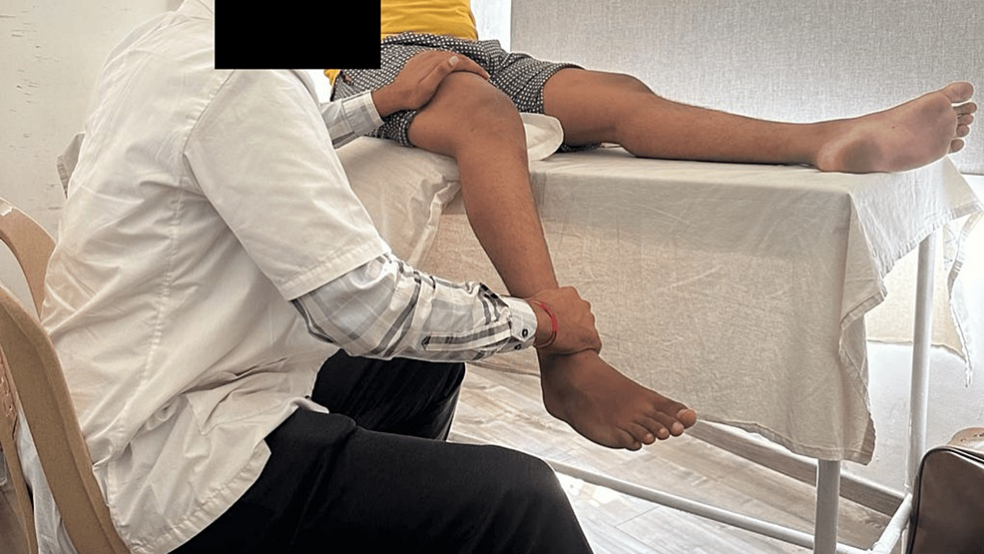
Patient being treated by performing myofascial release technique

The physiotherapy treatment that was applied to the patient is mentioned in Table 1. Figure 2 shows the patient being treated with the help of the physiotherapist.

**Table 1:** Physiotherapy treatment ROM: range of motion, SLR: straight leg raise, VMO: vastus medialis obliques, reps: repetition, MET: muscle energy technique, sec: second

GOAL	INTERVENTION	RATIONALE
Patient education and counselling	It informs the patient about the importance of exercising and the need for precaution.	Developing the confidence of patients to increase the efficacy of treatment. Educating on the necessity of precaution to prevent further deterioration of the condition.
	Precautions include avoiding strenuous activities, sprinting and running.	
To manage pain and swelling	On the site of injury cryotherapy (15-20 minutes every 3 hours)	Reduce pain and swelling
To maintain mobility of distal and proximal joints, to increase the muscle strength, to maintain ROM	General mobility exercises include SLR (10 reps), heel slides (10 reps), VMO strengthening (5 sec hold x 10 reps), hip extensors strengthening in prone (10 sec hold x 10 reps), MET for quadriceps (5 sec x 10 reps), dynamic quadriceps using quadriceps chair (5 sec hold x 10 reps), mini squats (10 reps), posterior capsule stretch by keeping pillow roll under the heel (30 sec x 5 reps), cycling for 5 mins.	Strengthening of hip flexor and extensor muscles of unaffected and affected limb. Helps to maintain and improve the ROM of the proximal and distal joints. Helps in improving the extensibility (lengthening) of the muscles.
Balance and proprioceptive training	Leg press against Swiss ball with hip and knee in 90-degree position (20 sec x 10 reps)	Helps to maintain and improve joint approximation and joint position by giving proprioceptive training; thus, it improves balance and weight bearing on the affected limb and reduces further risk of instability and injury.
To maintain skin integrity and mobility	Scar tissue mobilisation (vertically and horizontally)	Helps to maintain the integrity of the skin. Preventing and breaking adhesion formation.
To maintain patellar alignment and stability of the joint	Long knee brace, patellofemoral joint mobilization	Helps in maintaining the stability and mobility of the joint.

## Discussion

This case report illuminates a prevalent scenario in sports-related injuries, notably in cricket, where swift movements and sudden impacts can precipitate patellar dislocations. The individual in question, a 19-year-old cricket player, encountered recurrent patellar instability, prompting the diagnosis of a ruptured MPFL and the recommendation for arthroscopic reconstruction. The findings from the assessment and examination yield valuable insights into the extent of the injury. The discernible scar on the medial knee aspect signifies a prior surgical intervention, potentially indicative of a previous episode of patellar dislocation. The reported pain levels, gauged using the VAS, underscore the profound impact of the injury on the patient's daily activities and overall quality of life (QOL). The instability and locking further emphasize the gravity of the MPFL tear and its notable influence on the patient's ambulatory abilities and functional prowess. These manifestations strongly indicate the imperative for intervention to rectify the underlying anatomical aberration and reinstate stability to the patellofemoral articulation [15]. The decision to opt for arthroscopic reconstruction aligns with the prevailing standard of care for cases involving MPFL tears associated with recurrent patellar instability. Arthroscopic methodologies afford precise visualization and repair of the compromised ligament, curtailing surgical invasiveness and expediting recovery.

In the postoperative phase, the focal point of an integrated rehabilitation approach assumes paramount importance [16]. Physiotherapy is pivotal in reinstating patellar stability, augmenting the range of motion, and fortifying the surrounding musculature, including the VMO muscle [11]. The postoperative rehabilitation regimen following MPFL reconstruction should be rooted in a comprehensive comprehension of lower limb biomechanics and anatomical considerations, coupled with a deep understanding of the surgical intervention. Also, it is necessary to thoroughly assess the patient to tailor the program effectively [16,17]. Patients who underwent MPFL reconstruction and aimed to resume sporting or functional activities followed a structured physical therapy regimen. This program incorporated proprioceptive-focused and dynamic rehabilitation elements, complemented by a home exercise regimen. Encouraged by the favorable outcomes of this approach and a thorough review of pertinent literature concerning MPFL rehabilitation, a carefully designed rehabilitation protocol was introduced [15].

This all-encompassing rehabilitation regimen is meticulously devised to optimize the patient's recovery and facilitate a return to pre-injury functional capacities. Moreover, this case report underscores the criticality of tailored treatment strategies. Consideration of each patient's anatomical and biomechanical nuances and specific athletic requisites is imperative in effectively customizing the rehabilitation program.

## Conclusions

This case report demonstrates the effectiveness of a comprehensive strategy encompassing surgical intervention MPFL reconstruction and subsequent post-operative rehabilitation for addressing recurrent patellar instability stemming from an MPFL tear. It underscores the importance of meticulous evaluation, personalized treatment strategy, and cooperative efforts between orthopedic surgeons and physiotherapists to attain a favorable outcome. Moreover, it underscores the imperative for continual research and clinical investigations to advance and optimize treatment protocols for analogous cases in forthcoming medical practice.
